# Return to Athletic Activity Following Sesamoidectomy: A Systematic Review of Time Frames and Clinical Outcomes

**DOI:** 10.1177/23259671261477555

**Published:** 2026-08-26

**Authors:** Nour-Maria Bouzid, Mohini Johri, Claire Simpson, Benjamin P. Cassidy, Andrew S. Cuthbert, Joshua Setliff, John W. Cyrus, Christopher D. Murawski, Annunziato Amendola, Conor N. O'Neill

**Affiliations:** † Virginia Commonwealth University School of Medicine, Richmond, Virginia, USA; ‡ Department of Orthopaedic Surgery, Virginia Commonwealth University Health System, Richmond, Virginia, USA; § Department of Research and Education, Virginia Commonwealth University, Richmond, Virginia, USA; ‖ Duke Department of Orthopaedic Surgery, Duke University Health System, Durham, North Carolina, USA; Investigation performed at Virginia Commonwealth University School of Medicine, Richmond, Virginia, USA

**Keywords:** sesamoidectomy, hallux sesamoid bones, sport, return to sport

## Abstract

**Background:**

Hallux sesamoid pathology can cause persistent forefoot pain and loss of push-off strength in athletically active individuals, and sesamoidectomy is commonly performed when prolonged nonoperative management fails.

**Purpose:**

To evaluate return to athletic activity, return to preoperative performance, and postoperative complications following partial or complete hallux sesamoidectomy in athletically active individuals.

**Study Design:**

Systematic review; Level of evidence, 4.

**Methods:**

PubMed, Embase, and CENTRAL were searched through October 2025 for studies reporting outcomes in athletically active individuals undergoing sesamoidectomy. Data extraction included demographics, pathology, operative details, follow-up duration, return-to-sport metrics, return to preoperative performance, and complications.

**Results:**

Six studies met inclusion criteria, representing 193 patients. Female patients comprised 72.2% of reported cases. The weighted mean return-to-sport rate was 91.9%. The weighted mean time to return to sport was 12.2 weeks. Return to preoperative performance occurred in 83.6% of patients. Complication rates ranged from 0% to 29.2%, with a weighted mean complication rate of 12.5%.

**Conclusion:**

Sesamoidectomy allows most athletically active individuals to return to athletic activity within 3 to 4 months postoperatively, although approximately 16% fail to return to their preoperative level of performance. Postoperative complications occurred in 12.5% of patients and should be considered during preoperative counseling. These findings may help guide shared decision-making for patients considering sesamoidectomy after failed conservative treatment.

**Registration:**

CRD420251170096 (PROSPERO).

The hallux sesamoid bones are contained in the insertion of the flexor hallucis brevis and form part of the plantar plate of the first metatarsophalangeal joint, functioning to absorb load and generate efficient push-off during gait and sport.^6^ Sesamoidectomy is a surgical option for persistent hallux sesamoid pathology, most commonly chronic sesamoiditis, fracture/nonunion, or osteonecrosis, when prolonged conservative management fails and pain continues to limit function.^1,12^ In athletically active individuals, symptoms in the sesamoid complex are especially problematic because the sesamoids contribute to first metatarsophalangeal joint load sharing and the plantarflexion moment at toe-off, making sprinting, cutting, and jumping painful or mechanically compromised.^6^ As a result, operative excision of part or all of the involved sesamoid is frequently considered when persistent symptoms prevent return to athletic activity despite rest, immobilization, orthotics, or activity modification.^2,3,5,8,11,12^

Patients considering surgical intervention benefit from specific expectations regarding return to sport (RTS) participation and the likelihood of returning to their preoperative level of performance.^14^ However, published reports describing sesamoidectomy in athletic or athletically active cohorts vary in how RTS is defined and measured, and there often is not a clear distinction between return to participation in sports and return to preoperative performance level.^1,11,12^ Prior systematic reviews, including a 2018 review by Shimozono et al,^13^ have evaluated outcomes after sesamoidectomy; 4 of the 6 studies included in the present review^1,2,8,12^ were also included in that analysis, but the review did not incorporate more recent cohorts published after 2018, including Saxena et al^11^ and Dean et al.^5^ A contemporary review focused on RTS metrics and postoperative complications in athletically active individuals remains necessary to support preoperative counseling and shared decision making by providing realistic expectations regarding timing of RTS and the likelihood of achieving prior performance level.

Given this knowledge gap, we sought to clarify postoperative expectations for athletically active individuals after sesamoidectomy. The primary aim of this study was to synthesize outcomes after partial or complete hallux sesamoidectomy, with an emphasis on RTS, performance recovery, and postoperative complications.

## Methods

### Literature and Database Search

**Figure 1.**
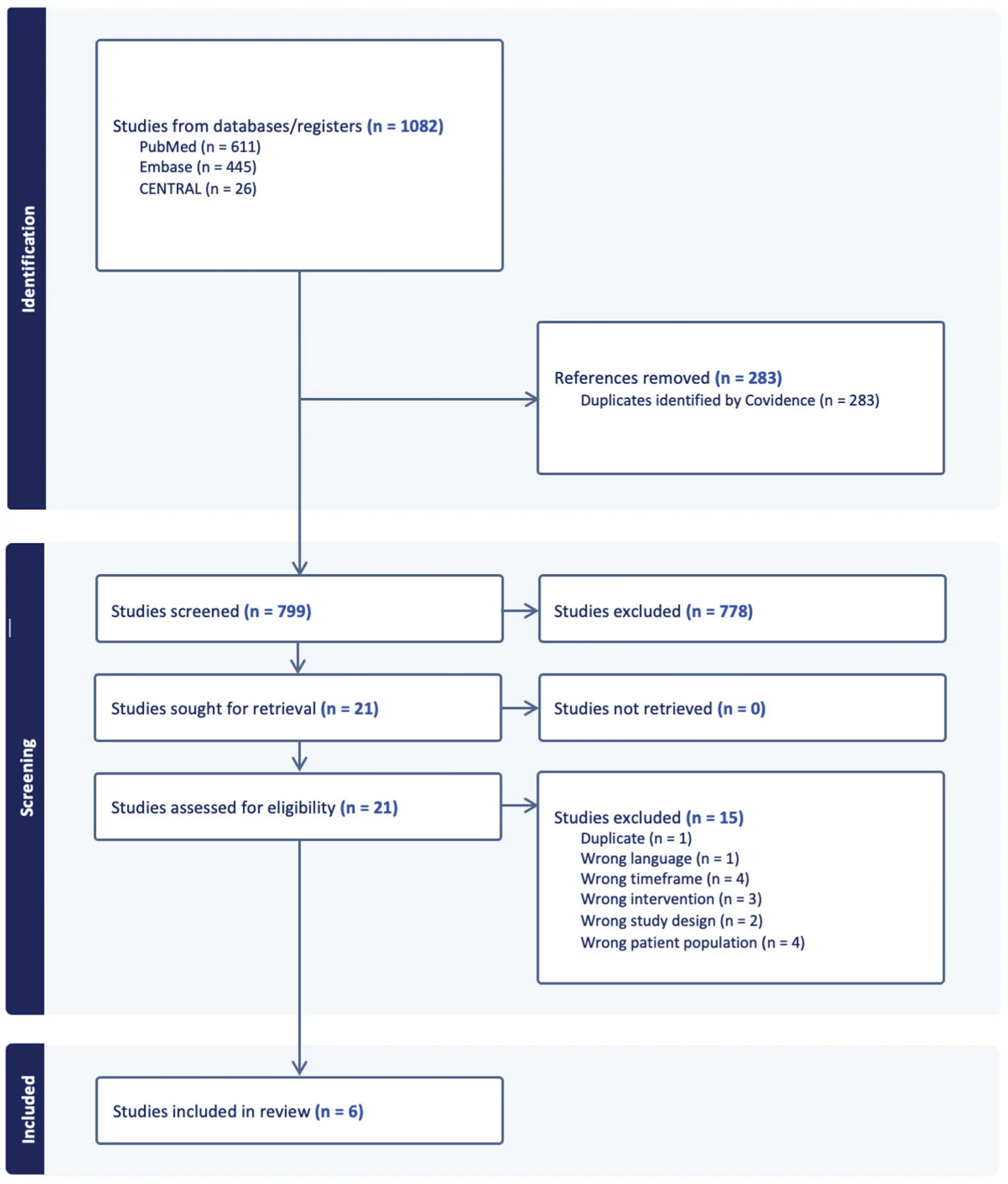
PRISMA (Preferred Reporting Items for Systematic Reviews and Meta-Analyses) flow diagram of study identification, screening, eligibility, and inclusion.

A systematic search of peer-reviewed, published literature was conducted in consultation with a research librarian (J.C.) in PubMed, Embase, and CENTRAL databases from January 2000 through October 2025. The search was conducted with the following terms and controlled vocabulary: “sesamoidectomy”, “hallux sesamoid”, “return to sport”, and “athletes” (Table 1). The results of the search in PubMed, Embase, and CENTRAL were imported into Covidence software for screening, as shown in Figure 1.^4^ The literature search and subsequent review were conducted in accordance with PRISMA (Preferred Reporting Items for Systematic Reviews and Meta-Analyses) reporting standards.^10^ The review protocol was registered in PROSPERO.

**Table 1:** Full Literature Search Strategy

Database	Search Date	Search Strategy	Hits
Embase	10/16/2025	1. (sesamoidectomy or surgical or surgery or operative or excision or resection or treatment).ti,ab,kf. or sesamoid bone/su 2. (sesamoiditis or sesamoid or sesamoids or &quot;great toe&quot; or hallux).ti,ab,kf. or exp hallux/ 3. ((return adj3 (sports or play or activity or activities or participation)) or (athlete or athletes or athletic or athletics or sports)).ti,ab,kf. or (exp &quot;return to sport&quot;/ or exp sport/ or exp athlete/) 4. 1, 2, and 3	445
PubMed	10/16/2025	1. ((sesamoidectomy OR surgical OR surgery OR operative OR excision OR resection OR treatment OR &quot;Sesamoid Bones/surgery&quot;[MeSH]) AND (sesamoiditis OR sesamoid OR sesamoids OR &quot;great toe&quot; OR hallux OR &quot;Hallux&quot;[MeSH])) AND (&quot;return to sport&quot;[MeSH Terms] OR &quot;return sport&quot;[Title/Abstract:~2] OR &quot;return sports&quot;[Title/Abstract:~2] OR &quot;return play&quot;[Title/Abstract:~2] OR &quot;return activity&quot;[Title/Abstract:~2] OR &quot;return activities&quot;[Title/Abstract:~2] OR &quot;return participation&quot;[Title/Abstract:~2] OR athlete[tiab] OR athletes[tiab] OR athletic[tiab] OR athletics[tiab] OR sports[tiab] OR &quot;Sports&quot;[MeSH] OR &quot;Athletes&quot;[MeSH])	611
CENTRAL	10/16/2025	1. (sesamoidectomy OR surgical OR surgery OR operative OR excision OR resection OR treatment):ti,ab,kw OR [mh &quot;Sesamoid Bones&quot;] 2. (sesamoiditis OR sesamoid OR sesamoids OR &quot;great toe&quot; OR hallux):ti,ab,kw OR [mh &quot;Hallux&quot;] 3. [mh &quot;return to sport&quot;] or [mh &quot;sports&quot;] OR [mh &quot;athletes&quot;] or (&quot;return to sport&quot; or &quot;return to sports&quot; or &quot;return to play&quot; or &quot;return to activity&quot; or &quot;return to activities&quot; or &quot;return to participation&quot; or athlete or athletes or athletics or sports):ti,ab,kw 4. 1, 2, and 3	26

### Eligibility Criteria

Studies were deemed eligible for inclusion if they examined athletically active individuals undergoing partial or complete hallux sesamoidectomy and reported ≥1 RTS-related metric, including RTS rate, time to RTS or athletic activity, return to preoperative performance rate, or time to return to preoperative performance. An athletically active individual was defined broadly to include individuals participating in organized or regular sport-related activity at the recreational, competitive, collegiate, or professional level. Given the limited number of studies, nonrandomized designs were included. Nonhuman, cadaveric, or biomechanical studies were excluded, as were non-English publications. Letters, editorials, technical notes, and surgical technique papers without clinical outcomes were also excluded, along with nonoperative cohorts. Studies in which sesamoidectomy outcomes could not be separated from outcomes of other procedures were excluded (Table 2).

**Table 2:** Inclusion and Exclusion Criteria

Inclusion Criteria	Exclusion Criteria
Studies on athletically active individuals undergoing partial or complete hallux sesamoidectomy reporting ≥1 return-to-sport metric	Nonhuman or cadaveric/biomechanical studies Non-English studies Letters, editorials, and technical or surgical technique papers without clinical outcomes Nonoperative cohorts Studies where sesamoidectomy outcomes cannot be separated from other procedures

### Study Selection and Data Extraction

The reviewer team consisted of medical student reviewers trained in systematic review methodology, with oversight from an attending orthopaedic foot and ankle surgeon (C.N.O.). Each study was screened by 2 independent reviewers (N.-M.B. and M.J.) using Covidence software.^4^ Disagreements at the title/abstract stage were resolved by a third reviewer (C.S.), and the same process was used for full-text screening. For included studies, the 2 initial reviewers independently extracted data onto a shared Google Sheets database (Google LLC). Reviewers cross-checked each other's entries against the original manuscripts, resolved discrepancies by discussion, and referred unresolved issues to the third reviewer for adjudication. Studies from the same author group were included only after confirming clear evidence of nonoverlapping cohorts.

For each included study, we recorded study characteristics, patient demographics, operative details, underlying pathology, and postoperative outcomes. Study characteristics included study design, level of evidence, and sample size. Patient characteristics included age, sex distribution, athletic participation level, and follow-up duration. Operative details included whether the medial or lateral sesamoid was excised and the surgical approach. Outcomes of interest included RTS or athletic activity, time to RTS, return to preoperative performance level, overall complication rates, and reported postoperative complications.

### Risk-of-Bias Assessment

Methodological quality and risk of bias were evaluated using the Joanna Briggs Institute (JBI) critical appraisal checklists, one for cohort studies and one for case series, based on each study's design.^9^ These tools use structured questions to judge the rigor of the study methods, the appropriateness of the analyses, and the clarity of how results are reported. The 2 independent reviewers appraised all included studies, discussed any differences to reach agreement, and the third reviewer adjudicated if consensus could not be reached.

### Statistical Analysis

Categorical variables were summarized as counts and proportions. Continuous variables were summarized using weighted means, with each study weighted by the number of patients contributing data to that endpoint. Weighted means and proportions were calculated by multiplying each study estimate by the corresponding sample size, summing across studies, and dividing by the total number of patients contributing data for that outcome. When direct comparisons were made between outcome measures, *P* values were reported to determine any statistically significant difference between the 2 groups. Each outcome-specific synthesis included only studies reporting the corresponding outcome of interest. Outcomes of interest were tabulated to present study-level results and facilitate comparison across individual studies. A formal meta-analysis was not performed because of substantial clinical and methodological heterogeneity across the included studies. Although the studies evaluated sesamoidectomy outcomes, there was marked variability in patient populations, study design, surgical indications, outcome definitions, follow-up duration, and reporting of RTS and complication metrics. Several studies did not report variance measures or standardized outcome instruments, and key endpoints were inconsistently defined (eg, RTS vs return to preinjury performance). Additionally, the predominance of small, retrospective case series with uneven reporting limited the reliability of pooled effect estimates. In accordance with established systematic review methodology, we determined that quantitative synthesis would risk generating misleading summary statistics and instead performed a structured qualitative synthesis.

## Results

### Literature Search and Screening

The database search identified 1082 records. After removal of 283 duplicates, 799 studies remained for title and abstract screening. Of these, 778 were excluded based on the prespecified criteria, leaving 21 studies for full-text review; all full texts were successfully retrieved. Following full-text screening, 15 studies were excluded (duplicate, wrong language, wrong time frame, wrong intervention, wrong study design, or wrong patient population), resulting in 6 studies included for data extraction^1,2,5,8,11,12^ (Figure 1). Study identification, screening, and reporting were performed in accordance with PRISMA guidelines.^10^

### Risk-of-Bias Assessment

The 2 independent reviewers assessed methodological quality and risk of bias for each included study using the appropriate JBI critical appraisal checklist based on study design (cohort study or case series).^9^ The 5 case series^1,2,5,11,12^ were level 4 evidence, and the 1 retrospective cohort study^8^ was level 3 evidence. The single cohort study received a score of 9 out of 11. The 5 case series had a mean score of 9.8 out of 10. Based on these appraisals, all studies were considered sufficiently rigorous to be included in this review (Appendix Table A1).

### Patient Demographics

The 6 included studies reported outcomes for a total of 193 patients who underwent hallux sesamoidectomy (Table 3). Sex distribution was reported in 5 studies (n = 169),^2,5,8,11,12^ comprising 122 female (72.2%) and 47 male (27.8%) patients. Mean age was reported in all studies and ranged from 16.8 to 35.4 years with a weighted mean age of 30.9 years. The study population included athletically active individuals across a spectrum of competition levels, ranging from recreational participation to collegiate and professional sport, and represented a variety of activities including football, track and field, dance, gymnastics, tennis, aerobics, and soccer.

**Table 3:** Study Characteristics and Outcomes After Hallux Sesamoidectomy*^*a*^*

Study (year)	Study Design (LOE)	Total N	Sex	Mean Age ± SD, y	Medial or Lateral Sesamoid Excision	Mean Follow-Up Duration, mo	RTS, %	Mean Time to RTS, wk	Return to Preoperative Performance, %	Diagnosis	Complication Rate, %
Saxena et al (2022)	Case series (4)	68	44 F 24 M	28.4 ± 12.2	29 lateral 41 medial	106.6 ± 66.6	–	11.1 ± 5.1	–	–	5.7
Dean et al (2020)	Case series (4)	26	25 F 1 M	27.2 ± 14.8	5 lateral 20 medial 1 both	–	80.0	20.1 ± 4.39	67.0	18 osteochondrosis, 4 chronic sesamoiditis, 3 fracture nonunion, 1 bipartite sesamoid	–
Kane et al (2017)	Retrospective cohort study (3)	46	Lateral: 17 F 7 M Medial: 10 F 12 M (*P* = .13)	Lateral: 36 ± 14.5 Medial: 34 ± 16.2 (*P* = .58)	24 lateral 22 medial	Lateral: 58.3 ± 31 Medial: 36.7 ± 62.8 (*P* = .61)	97.8	Lateral: 10.2 ± 2.9 Medial: 12.7 ± 4.3 (*P* = .16)	Lateral: 91.7 Medial: 95.5 (*P*≥ .99)	All fractures (type unspecified)	Lateral: 29.2 Medial: 18.2 (*P* = .50)
Bichara et al (2012)	Case series (4)	24	–	32.2 ± 10.4	9 lateral 15 medial	35 ± 21	91.6	11.6 ± 3.87	–	All fracture nonunion	8.3
Saxena and Krisdakumtorn (2003)	Case series (4)	24	21 F 3 M	35.4	10 lateral 16 medial	86.4	–	Athletes: 7.5 Athletically active: 12.0 (*P* < .02)	83.3	–	16.7
Biedert and Hintermann (2003)	Case series (4)	5	5 F	16.8	5 medial	50.6	100	8	80.0	All stress fractures	0.0

a Data are presented as mean or mean ± SD unless otherwise indicated. Dashes in table cells indicate data not reported. F, female; LOE, level of evidence; M, male; RTS, return to sport.

Follow-up duration was variable across studies (Table 3). Among the 5 studies that provided a value, follow-up ranged from 35.0 to 106.6 months with a weighted mean follow-up of 75.6 months.

Athletic participation level was stratified in only 2 studies. Bichara et al^1^ reported 5 intercollegiate athletes and 19 athletically active individuals. Saxena and Krisdakumtorn^12^ reported 11 athletes and 13 active individuals; athletes were defined as those competing at the professional, college, or high school varsity level, running >25 miles per week, or participating in sport activity for >6 hours per week. The remaining studies described participants as athletes or athletically active individuals and reported sports participation, but did not further classify competition level.

Diagnosis-specific pathology was reported in 4 included studies (Table 3)^1,2,5,8^. Reported indications for sesamoidectomy included sesamoid fractures of unspecified type (n = 46), fracture nonunions (n = 27), stress fractures (n = 5), osteochondrosis (n = 18), chronic sesamoiditis (n = 4), and bipartite sesamoid (n = 1).

### RTS and Performance Recovery

RTS was reported in 4 of the 6 included studies (Table 3).^1,2,5,8^ Across these studies, the weighted mean RTS rate was 91.9%. Individual study RTS rates ranged from 80% to 100% among reporting cohorts, although definitions of RTS and the level of sport at return varied by study.

Time to RTS was reported in all 6 studies (Table 3), with a weighted mean time to RTS of 12.2 weeks. Reported mean time-to-return values ranged from 8.0 to 20.1 weeks.

Return to preoperative performance (or return to maximal capacity) was reported in 4 of the 6 studies (Table 3).^2,5,8,12^ The weighted mean rate of return to preoperative performance was 83.6%. Across reporting studies, the proportion returning to preoperative performance ranged from 67% to 91.7%.

Data were not sufficiently available or consistently reported to analyze RTS rate, time to RTS, return to preoperative performance, or time to return to preoperative performance by surgical approach, laterality, diagnosis, sport type, competition level, foot alignment, or complication status.

### Postoperative Complications

Complications were reported in all 6 studies, with 5 studies providing a quantifiable complication rate (Table 3).^1,2,8,11,12^ Across those 5 cohorts, complication rates ranged from 0% to 29.2%, with a weighted mean complication rate of 12.5%. Reported complications included neurologic complications, including numbness and neuroma-like symptoms, which were the most common (28.6%). Inflammatory or pain-related complications followed, including transient sesamoiditis of the remaining sesamoid (19.0%) and metatarsalgia or flexor hallucis tenosynovitis (4.8%). Alignment or mechanical complications included hallux deformity such as hallux valgus or hallux varus (14.3%), first metatarsophalangeal joint stiffness (9.5%), and functional weakness with loss of hallux flexion (4.8%). Soft tissue complications, including wound dehiscence or superficial infection, occurred in 9.5% of complications, while thrombotic complications, specifically deep vein thrombosis, were also reported in 9.5%.

Complication-specific return outcomes were reported in 2 included studies.^1,12^ In Bichara et al,^1^ the 2 patients who failed to return to activity experienced symptomatic hallux valgus and metatarsalgia/flexor hallucis longus tenosynovitis. In Saxena and Krisdakumtorn,^12^ patients with hallux valgus and hallux varus returned to activity at 12 weeks, while the 2 patients with neuritis/nerve entrapment returned at 10 and 16 weeks, respectively. Return to preoperative performance outcomes according to complication status were not reported in any studies.

Among studies with laterality-specific data, medial complication rates ranged from 0% to 18.2% and lateral complication rates ranged from 6.9% to 30.0%, with weighted mean complication rates of 8.5% and 18.1%, respectively (Table 4).^1,8,11,12^ No complications were reported by Biedert and Hintermann,^2^ and Dean et al^5^ did not report laterality-specific complication data.

**Table 4:** Laterality-Specific Complications Following Sesamoidectomy*^*a*^*

Study	Medial Sesamoidectomy Complications, n	Medial Sesamoidectomy Complication Rate, %	Lateral Sesamoidectomy Complications, n	Lateral Sesamoidectomy Complication Rate, %	Surgical Approach
Saxena et al (2022)	Deep vein thrombosis (2)	4.9	Wound dehiscence (1) Weakness with hallux flexion (1)	6.9	Medial: direct medial Lateral: direct plantar
Dean et al (2020)*^*b*^*	–	–	–	–	Medial: medial Lateral: plantar
Kane et al (2017)	Transient sesamoiditis (2) Stiff MTP joint (1) Superficial infection at incision site (1)	18.2	Transient sesamoiditis (2) Stiff MTP joint (1) First webspace numbness (4)	29.2	Medial: midaxial Lateral: combined medial/web-space → direct plantar*^*c*^*
Bichara et al (2012)	Hallux valgus (1)	6.7	Pain related to metatarsalgia and flexor hallucis tenosynovitis (1)	11.1	Medial: medial Lateral: dorsal
Saxena and Krisdakumtorn (2003)	Hallux valgus (1)	6.3	Hallux varus (1) Scarring with neuroma-like symptoms (2)	30.0	Medial: 4 medial, 12 dorsomedial Lateral: 3 plantolateral, 7 dorsolateral
Biedert and Hintermann (2003)	None	0.0	–	–	Medial: medial

a Dashes in table cells indicate data not reported. MTP, metatarsophalangeal.

b Complications were not specific to the athletically active cohort.

c Arrow indicates transition to a different approach.

## Discussion

This review found that sesamoidectomy is associated with a return to athletic participation in >90% of patients within 3 months and that return to preoperative performance level is variable, with >16% of athletically active individuals failing to return to preoperative performance. Although RTS outcomes were generally favorable, complications occurred in a meaningful proportion of patients and represent an important consideration when weighing the potential benefits of surgery against its associated risks. Hallux sesamoid pathology directly compromises toe-off mechanics and propulsion, prompting patients and surgeons to make a decision based on a narrow set of practical concerns: whether surgery can restore sport participation, how long return will take, and whether the patient can return at the same level. In that context, the benefits of a high RTS rate and a roughly 3-month return time frame should be weighed against the risks of postoperative complications when counseling patients who remain limited after prolonged conservative management.

Complication profiles differed across studies. Specifically, Kane et al^8^ reported that all perioperative complications were transient and resolved without long-term sequelae or reoperation, whereas Saxena and Krisdakumtorn^12^ described a small number of structural complications, including hallux varus and loss of hallux flexion, both of which required subsequent surgical intervention, while neurologic complications resolved with steroid injections. Limited complication-specific outcome data were available; however, in Saxena and Krisdakumtorn, patients experiencing postoperative complications returned to activity at a mean of 12.5 weeks compared with 11.1 weeks for the overall cohort, suggesting that complications may delay recovery in some patients. Conversely, Bichara et al^1^ reported that the 2 patients who failed to return to activity experienced postoperative complications. Although several reported complications were transient or successfully managed, the available evidence suggests that postoperative complications may contribute to delayed return to activity or failure to RTS in some patients.

A systematic review published in 2018 by Shimozono et al^13^ evaluated outcomes after sesamoidectomy for hallux sesamoid disorders in a broader population not limited to athletic patients. The authors reported that 94.4% of patients returned to sports, with 90.0% returning to their previous level at a mean of 11.8 ± 1.8 weeks; the overall complication rate was 22.5% (10 studies; 196 feet).^13^ In our synthesis, the weighted mean RTS rate (91.9%) and weighted mean time to RTS (12.2 weeks) were similar, consistent with return to activity commonly occurring around 3 months after surgery. Our weighted mean rate of return to preoperative performance (83.6%) was lower than the rate reported by Shimozono et al, which likely reflects differences in how performance is defined and assessed in more recent studies, as well as the higher functional demands typically implied by athletically active cohorts. It is also important to note that RTS generally captured resumption of athletic participation, whereas return to preoperative performance reflected recovery to prior level of play or maximal capacity. Complication rates also differed: while Shimozono et al reported an overall complication rate of 22.5%, the weighted mean complication rate across studies reporting this endpoint in our review was 12.5% (range, 0%-29.2%), suggesting that complication estimates are sensitive to cohort composition, follow-up duration, and how complications are captured and reported. Follow-up duration in the present review was longer than in Shimozono et al’s review, with a weighted mean of 75.6 months compared with 45.1 ± 19.3 months, which likely contributed to differences in reported outcomes.

Interestingly, sex distribution was heavily skewed toward female patients (72.2%), which was similarly noted in a study by Hardt et al.^7^ This may reflect sport exposures, bone health, morphology, biomechanics, or footwear-related factors, though current literature does not clearly identify a specific causation. Future work that reports sex-stratified incidence, indications for surgery, and RTS outcomes across comparable sports and competition levels would help clarify whether this pattern reflects true differences in risk, differences in treatment pathways, or differences in reporting.

Medial versus lateral sesamoid excision may differentially affect hallux biomechanics, including alignment, load sharing, and push-off mechanics. In the present review, laterality-specific complication reporting suggested a greater complication burden after lateral sesamoidectomy than after medial sesamoidectomy, with all 4 studies^1,8,11,12^ permitting direct within-study comparison demonstrating higher complication rates after lateral excision. However, the small number of studies with laterality-stratified data, inconsistent reporting across cohorts, and absence of formal subgroup analysis precluded definitive comparison.

Surgical approach and foot alignment may also influence outcomes after sesamoidectomy. Across the included studies, medial sesamoidectomy was consistently performed through a medial or dorsomedial approach, whereas lateral sesamoidectomy was most commonly performed through plantar or plantolateral approaches, although dorsal and combined approaches were also reported. Saxena and Krisdakumtorn^12^ observed shorter return-to-activity times following plantolateral versus dorsolateral approaches for lateral sesamoidectomy (7.3 vs 9.3 weeks) and following medial versus dorsomedial approaches for medial sesamoidectomy (10.0 vs 12.5 weeks); however, no statistical comparisons were performed because of the small sample size.^12^ Notably, 1 patient undergoing a dorsolateral approach developed hallux varus, and 2 patients undergoing a dorsolateral approach developed postoperative scarring and neuromas. Dean et al^5^ reported no apparent association between cavus or planovalgus foot type and sesamoid pathology, suggesting that pathology may be more closely related to sport-specific demands than underlying foot morphology. Similarly, Kane et al^8^ found statistically significant but clinically insignificant differences in hallux valgus and intermetatarsal angles between medial and lateral sesamoidectomy cohorts. Although these findings do not support a clear relationship between foot alignment and outcomes, they highlight operative and biomechanical factors that warrant further investigation in future studies.

### Limitations

This study is limited by the small body of literature on sesamoidectomy in athletically active individuals, with only 6 studies meeting inclusion criteria. In addition, study designs and outcome definitions were not uniform, particularly for RTS and return to preoperative performance, which limits direct comparisons across cohorts. Postoperative rehabilitation protocols were also variably reported across studies, which likely contributed to the observed variability in RTS timing (8-20 weeks). Last, several potentially important variables were inconsistently reported or not reported, including sport subtype, competition level (recreational, collegiate, or professional), sport-specific outcomes, foot alignment characteristics, pathology subtype, operative details, and complication definitions, which restricted synthesis across studies and precluded meaningful subgroup analyses.

## Conclusion

Sesamoidectomy allows most athletically active individuals to return to athletic activity within 3 to 4 months postoperatively, although approximately 16% fail to return to their preoperative level of performance. Postoperative complications occurred in 12.5% of patients and should be considered during preoperative counseling. These findings may help guide shared decision-making for patients considering sesamoidectomy after failed conservative treatment.

## Appendix

**Appendix Table A1:** Critical Appraisal Scores Based on Joanna Briggs Institute Checklists

Study	Score
Saxena et al (2022)^11^	10/10
Dean et al (2020)^5^	10/10
Kane et al (2017)^8^	9/11
Bichara et al (2012)^1^	10/10
Saxena and Krisdakumtorn (2003)^12^	9/10
Biedert and Hintermann (2003)^2^	10/10
